# Misophonia: physiological investigations and case descriptions

**DOI:** 10.3389/fnhum.2013.00296

**Published:** 2013-06-25

**Authors:** Miren Edelstein, David Brang, Romke Rouw, Vilayanur S. Ramachandran

**Affiliations:** ^1^Department of Psychology, Center for Brain and Cognition, University of California, San DiegoSan Diego, CA, USA; ^2^Department of Psychology, Northwestern UniversityEvanston, IL, USA; ^3^Department of Psychology, University of AmsterdamAmsterdam, Netherlands

**Keywords:** misophonia, sound sensitivity, skin conductance response, auditory processing, aversive sounds, case reports, autonomic response

## Abstract

Misophonia is a relatively unexplored chronic condition in which a person experiences autonomic arousal (analogous to an involuntary “fight-or-flight” response) to certain innocuous or repetitive sounds such as chewing, pen clicking, and lip smacking. Misophonics report anxiety, panic, and rage when exposed to trigger sounds, compromising their ability to complete everyday tasks and engage in healthy and normal social interactions. Across two experiments, we measured behavioral and physiological characteristics of the condition. Interviews (Experiment 1) with misophonics showed that the most problematic sounds are generally related to other people's behavior (pen clicking, chewing sounds). Misophonics are however not bothered when they produce these “trigger” sounds themselves, and some report mimicry as a coping strategy. Next, (Experiment 2) we tested the hypothesis that misophonics' subjective experiences evoke an anomalous physiological response to certain auditory stimuli. Misophonic individuals showed heightened ratings and skin conductance responses (SCRs) to auditory, but not visual stimuli, relative to a group of typically developed controls, supporting this general viewpoint and indicating that misophonia is a disorder that produces distinct autonomic effects not seen in typically developed individuals.

## General introduction

Misophonia, literally translated to “hatred of sound,” is a chronic condition in which specific sounds provoke intense emotional experiences and autonomic arousal within an individual. Trigger stimuli include repetitive and social sounds typically produced by another individual, including chewing, pen clicking, tapping, and lip smacking. These experiences are not merely associative in nature, but drive the sufferer to avoid situations in which they may be produced, limiting one's ability to interact with others and often leading to severe problems in their social and professional lives. Also known as selective sound sensitivity syndrome, the term “misophonia” was first coined by Jastreboff (Jastreboff, 2000; Jastreboff and Jastreboff, 2001a,b, 2003) and little remains known about the condition. To our knowledge only two case studies (Hadjipavlou et al., 2008; Schwartz et al., 2011) and one clinical study (Schröder et al., 2013) have examined misophonia. In the latter study, psychiatrists presented questionnaires and administered interviews to 42 misophonics, an essential first step in showing that misophonia is a primary disorder with no obvious comorbidity with other known psychological or neurological conditions (Schröder et al., 2013).

The prevalence of misophonia is under active investigation but there exist several online support groups with thousands of members (Misophonia UK, Facebook and Yahoo). Sufferers of misophonia are fully aware of its presence and the abnormal responses they have to their trigger sounds. In addition, many sufferers have identified the condition in at least one close relative, suggesting a possible hereditary component. While effective treatments for misophonia remain elusive, individuals report utilizing coping mechanisms to minimize their exposure and response to triggering stimuli (discussed at length below). Further, misophonia appears to exhibit some general similarities to tinnitus. Jastreboff and Hazell (2004) propose that misophonia and tinnitus are both associated with hyperconnectivity between the auditory and limbic systems, suggesting that both conditions would evoke heightened reactions to their respective sounds. However, despite these general similarities, misophonia differs from tinnitus considerably, particularly in terms of how the condition is localized around certain human-produced sounds and situations as opposed to internally perceived, abstract sounds.

While the majority of typically developing individuals experience general and unelaborated emotional reactions to a range of sounds (Halpern et al., 1986), these widespread negative associations remain non-debilitating and at most an annoyance to the listener. One critical possibility is that the valenced associations present in typically developing individuals are matched to those with misophonia, with the latter merely experiencing a more extreme physiological response. Indeed, the sound of fingernails on a chalkboard is an emotionally evocative stimulus that elicits extreme discomfort in the typical population (Zald and Pardo, 2002; Kumar et al., 2012) and misophonic individuals often reference this stimulus in illustrating the extreme nature of their trigger sensations. In this study, we further elaborate on the symptoms and behaviors associated with misophonia as well as examine whether misophonics' physiological responses support their subjective reports of feeling autonomic arousal in response to certain sounds.

## Experiment 1

### Introduction

We first received information about misophonia in December of 2011 through members of an online misophonia support group. From initial descriptions, the condition appeared to have many intriguing qualities in addition to being quite unknown and unexplored. Misophonic individuals were invited to the lab for preliminary interviews with the hope of gaining a more concrete understanding of their experiences with the condition.

### Materials and methods

#### Participants

Eleven individuals with misophonia from the San Diego and Los Angeles areas were recruited from the University of California, San Diego campus, through self-identified contact of our lab as well as through an online misophonia support group (4 males and 7 females, mean age = 35.82; range = 19–65).

#### Procedure

Thirty to sixty minute semi-structured interviews were conducted by members of our research group on the University of California, San Diego campus. As no set diagnostic criteria for misophonia exists for misophonia, eligibility for study inclusion was based on severity of symptoms paired with experiential descriptions reported by the subject. The five initial interviews were exploratory in nature and included a range of topics, including approximate age of onset, lists of sounds that elicit varying degrees of discomfort, whether or not certain individuals exacerbate the condition, coping mechanisms, common thoughts when experiencing symptoms, physical responses to the trigger sounds, effect of the condition on their daily lives, and other potentially comorbid medical conditions. From these interviews we were able to generate a core set of questions to create the general framework of the subsequent six interviews that were held.

### Results and discussion

After conducting all 11 interviews, it was apparent that the experiences of the misophonics, though intrinsically variable between subjects, contained noticeable trends and similarities. The most salient categories of assessment and their traits are documented in Table 1. In addition, it should be noted that all diagnostic criteria listed by Schröder et al. (2013) were present in the reports of our misophonic subjects (see Table 1) even though these interviews were conducted prior to the publishing of that article.

**Table 1:** **Summary of qualitative data gathered from interviews of the 11 misophonic subjects (4 males and 7 females, mean age = 35.82; range = 19–65) in Experiment 1, broken down into 18 of the most salient diagnostic categories**.

Age of onset	8–10 years old (3)–27%
	As long as can remember (3)–27%
	Childhood (3)–27%
	17 (1)–9%
	Early teenage years (1)–9%
Worst trigger sounds	Eating/chewing/crunching sounds (11)
	Lip smacking (2)
	Pen clicking (2)
	Clock ticking (2)
Other trigger sounds	Low frequency bass sounds (8)
	Pen clicking (4)
	Footsteps (3)
	Finger tapping (3)
	Whistling sounds (3)
	Typing (3)
	Lip smacking (2)
	Clock ticking (1)
	Plastic bags (1)
	Repetitive barking (1)
	Finger tapping (1)
	Sniffling (1)
Localized around certain individuals?	Yes (9)–82%
	No (2)–18%
Worsened over time?	Yes (5)–45%
	Stays the same (3)–27%
	No, gotten better (2)–18%
	N/A (1)–9%
Own trigger sounds ok?	Yes (10)–91%
	Avoids producing own trigger sounds (1)–9%
Repetitive sounds worse
	Yes (9)–82%
	N/A (2)–18%
Runs in family?	Yes (6)–55%
	Not known (3)–27%
	N/A (2)–18%
Coping strategies	Avoiding or removing self from certain situations (7) (^*^D,E)
	Mimicry to “cancel out” sound or retaliate (6)
	Earplugs/headsets/music (6)
	Is conscientious about own sounds (5)
	Distract self (5)
	Ask others to stop (4)
	Positive internal dialog (1)
Effect of alcohol/caffeine	Alcohol lessens symptoms (7)
	Caffeine worsens symptoms (4)
	Symptoms not affected by caffeine (2)
	Does not use caffeine (2)
	Does not use alcohol (2)
	Symptoms not affected by alcohol (1)
	N/A (1)
Physical locations and descriptions of discomfort (^*^A)	Pressure in chest, arms, head, or whole body (5)
	Clenched/tightened/tense muscles (5)
	Increase in body temperature, blood pressure, or heart rate (2)
	Pained by trigger sounds (1)
	Hard to breathe (1)
	Sweaty palms (1)
Visual triggers	Jiggling/swinging legs (5)
Bothered by Ss sounds	Yes (6)–55%
	N/A (3)–27%
	No (2)–18%
Feelings and emotions associated with trigger sounds^*^	Sounds are invasive, intrusive, insulting, violating, offensive, disgusting, rude (9) (^*^A,D)
	Stress/anxiety (5)
	Anger or rage (4) (^*^D)
	Extreme annoyance/irritation (4) (^*^A,D)
	Panic (2) (^*^B)
	Impatience (1)
	Aggravation (1) (^*^D)
	Feeling trapped (1) (^*^B)
Other potentially comorbid medical conditions (^*^F)	Tinnitus (2)
	Obsessive-compulsive personality traits (2)
	Hyperacusis (1)
	Auditory processing disorder (1)
	ADD (1)
	PTSD (1)
	None (6)
Bothered by sounds produced by animals or children	Yes (1)–9%
	No (8)–73%
	N/A (2)–18%
Thoughts when experiencing trigger sounds	“I want to punch this person”
	“I hate this person”
	“Why won't they stop? I don't want to hurt their feelings by changing seats” (^*^C)
	“Why are they eating that way?”
	“Why are you doing that? It's rude”
	“Would you shut up?”
	“Stop it, I can't stand it”
	“Don't you know what you sound like?”
	“Why am I like this?” (^*^C)
	“Are they doing this on purpose?”
	“Why does he have to _____ so loudly?”
	“They should be more conscious of how they're affecting others”
	“I envy people who aren't bothered by sounds” (^*^C)
Effect on life	Realizes they are hyper focused on noises that should be in the background and are unable to ignore them (9) (^*^C,E)
	Cannot pay attention at a movie or in class when people are making trigger sounds (8) (^*^E)
	Tries not to be around people if they make trigger sounds (7) (^*^D,E)
	Can be triggered by sounds from television or videos (7) (^*^E)
	Triggers are worse when tired (7)
	Stays away from certain foods/avoids making certain sounds (3) (^*^D,E)
	Feels better when can locate source of sound (3)
	Thoughts of suicide (1)

*The number of subjects reporting a criterion can be found in parentheses to the right of each description*.

Criteria marked with an asterisk (

* *) designate diagnostic criteria (A–F) consistent with those proposed by Schröder et al. (2013)*.

*Please see General Discussion for more details*.

The most important criterion in misophonia is that particular sounds will evoke a disproportional aversive reaction. Our subjects were recruited based on their reports of this characteristic. In accordance with previous reports, our misophonics reported that the worst trigger sounds are chewing, eating, and crunching sounds, followed by lip smacking, pen clicking, and clock ticking (see Table 1). Other notable trigger sounds include low frequency bass sounds, footsteps, finger tapping, whistling sounds, and typing (see Table 1). Nine of our 11 misophonics reported that sounds repetitive in nature were particularly bad. In addition, six of our misophonics indicated that spoken “Ss” sounds were unpleasant, although not quite on the same level as trigger sounds.

In terms of aversive responses to these sounds, misophonics report a range of negative feelings, thoughts, as well as physical reactions. Some of the negative feelings experienced include intense anxiety, panic, anger, extreme irritation, and even rage (see Table 1). Additionally, in the context of our study, it is important to distinguish anxiety from fear. Specifically, while our subjects report feeling extreme stress and anxiety in response to trigger sounds, they did not report being afraid or fearful of them. Nine of our 11 misophonics reported trigger sounds as being invasive, intrusive, disgusting, or rude. They also reported feeling offended or violated by these sounds to the point where negative thoughts such as “I hate this person,” “Stop it, I can't stand it,” and “Don't you know what you sound like?” enter their minds. However, on top of the strong psychological effects, misophonics also report experiencing strong physical effects in response to trigger sounds. The most commonly reported physical effects were pressure in the chest, arms, head, or entire body as well as clenched, tightened, and tense muscles. Some misophonics reported an increase in blood pressure, heart rate or body temperature, sweaty palms, physical pain, and even difficulty breathing in response to trigger sounds (see Table 1). The aforementioned aversive responses evoked by trigger sounds are characteristic of a typical, autonomic nervous system response. In line with this, the worst situations for misophonics are often ones where they feel trapped and unable to escape, including long trips in cars or planes. Similarly, two misophonics report that trigger sounds at school or at home are worse than in places one can easily leave, such as a public place. However, despite extreme discomfort, misophonics generally do not physically act out on feelings of aggression. Some report instances of snapping at others while others internalize their frustration (see Table 1).

A final indication that misophonia produces physical and autonomic responses is the suggestion that pharmacological agents affect the condition. Four of our misophonics indicated that caffeine intensifies misophonic experiences while seven of misophonic individuals indicated that alcohol decreases symptomatology; these subjects describe that while under the influence of alcohol they can still hear the sound but their aversive response is not as strong.

In response to their aversive reactions to trigger sounds, misophonic individuals have developed a number of coping strategies including: avoiding or removing themselves from certain situations, mimicking trigger sounds, or the action producing it to “cancel out” or “retaliate,” utilizing earplugs, headsets or listening to music, distracting oneself, reciting positive internal dialog to help calm themselves, asking others to stop making the sounds, as well as being conscientious about their own sounds (see Table 1).

The degree to which quality of life is affected varied between our misophonic participants. One subject reported that misophonia “… does not affect the quality of my life too much. But it seems ridiculous and I would like to get rid of it” while another subject reported that misophonia had in the past evoked thoughts of suicide. These reports indicate there might be different degrees of the misophonic condition, ranging from mildly hindering to severely debilitating.

Misophonic individuals most commonly describe onset of the condition in childhood. Two subjects reported that with age, they learned to better cope with their misophonia, five subjects reported that it worsened over time (due to increasing aversiveness as well as increasing number of triggering stimuli) and three recalled no change over time. It is not fully understood why differences in trigger accumulation and severity develop between misophonics but it appears that prolonged and repeated exposure to a sound may be a contributing factor. For example, one of our misophonic subjects related this to the “honeymoon” period in a new job or relationship, in which for a few years new sounds caused little irritation. However, over time the negative affect of these sounds intensified to become triggers as well.

Six of our misophonics reported that one or several close family members display misophonic-like symptoms and behaviors. Two subjects had no information on this topic and three reported that they do not believe that misophonia runs in their families. While these reports are only anecdotal, they suggest there may be a familial or genetic component to misophonia, calling for further investigation in future studies.

Interestingly, misophonic individuals further report that responses evoked by trigger sounds appear to be modulated by prior knowledge, context, and sound source, implying that the condition is not driven simply by the physical properties of sound alone. For example, nine of our misophonics indicated that their misophonia is isolated to or exacerbated by certain individuals, usually close friends, coworkers, or family members whom they are exposed to frequently (see Table 1). Another curious characteristic described by 10 of our misophonics is the fact that self-induced trigger sounds (trigger sounds produced by the misophonic individual themselves) will not evoke nearly as much of an aversive response as when produced by others. In fact, as mentioned earlier, mimicking trigger sounds is one of the coping strategies utilized by misophonics to “overwrite” the disturbing sound being produced by another individual. Several misophonics even report eating foods in synchrony with the other person. However, mimicking is also mentioned as a way to retaliate against the offending individual producing the sounds, thus acting as a way to cope with the anger evoked by the condition.

The interviews further revealed an interesting effect of the role of context on aversive responses. For instance, eight of our misophonics report eating and chewing sounds (severely offensive triggers associated with rudeness when produced by human adults) will not bother them nearly as much if produced by animals or babies (see Table 1). One individual described that, as these individuals have little control over their actions and “don't know any better,” it helps in cancelling out strong aversive feelings. These results suggest that the aversive responses experienced by misophonics are explicitly tied to other individuals, implying an underlying social component to the condition. Accordingly, even though our subjects fit in with Schröder et al.'s (2013) diagnostic criterion of misophonics being aware of their condition, and recognizing their feelings as “excessive, unreasonable, or out of proportion,” they will still comment on the inappropriateness of another person's behavior nonetheless.

Another recurring topic from the interviews is the role of attention in misophonia. Nine of our misophonics report being hyper-focused on sounds that normally exist as background noise. One misophonic subject described the inability to tune out background noises as being like an “involuntary cocktail party effect” while another mentioned that “noises are never in the background. People sounds crash right through jet engine sounds.” Eight of our misophonics described being unable to pay attention to a movie or lecture when individuals around them produce trigger sounds, with partial remediation by distracting themselves and directing their attention elsewhere. In addition, it is possible that through understanding the role of attention in misophonia, potential treatments may be able to be developed.

In accordance with Schröder et al. (2013), our subjects reported a few symptoms shared with other diagnoses, however the complete symptomology of misophonia does not fit with any of the diagnostic categories in the diagnostic and statistical manual of mental disorders (DSM-IV). In their interviews, subjects described symptoms related to obsessive-compulsive disorder (OCD), attention deficit disorder (ADD), post-traumatic stress disorder (PTSD), auditory processing disorders as well as tinnitus and hyperacusis (see Table 1). However, these symptoms did not cover the full range of complaints, including the critical symptom of misophonia (a strong aversive response to particular sounds). Two of our misophonics reported being treated with medications, including antianxiety medications and antidepressants, that were intended to alleviate some of the effects of misophonia but as it stands, a treatment to fully address the root of the problem still remains elusive. Thus, our results are in line with the previous conclusion that misophonia is not part of another clinical, psychiatric, or psychological disorder (Schröder et al., 2013).

## Experiment 2

### Introduction

Qualitative assessments of misophonic subjects demonstrated the consistent association between specific sounds and intense emotional experiences. In order to confirm the presence of these emotional reactions and further examine their relationship to sound preferences present in the general population, we measured skin conductance response (SCR) while misophonic participants and typically developed individuals were exposed to aversive and non-aversive auditory, visual, and auditory-visual stimuli. SCR measures the electrical conductance of the skin and consequently the amount of sweat produced. Because sweat production is not under volitional control, SCR is widely accepted to indicate arousal of the sympathetic nervous system (Critcheley, 2002). For these reasons, we believe SCR to be an appropriate method of measuring autonomic arousal to various emotion-eliciting stimuli.

### Materials and methods

#### Participants

Six misophonic subjects who also participated in Experiment 1 (2 males and 4 females; mean age = 22.8; range = 19–30) and five controls (mean age = 22; range = 19–29) matched on age and gender participated in the experiment; A sixth control was excluded due to an error during data collection. Controls were recruited from the student population at the University of California, San Diego. All participants reported normal hearing and vision, gave signed, informed consent prior to the experiment, and participated either for cash or in fulfillment of a course requirement. The study was reviewed and approved by the university's Human Research Protections Program. Total experiment time was less than 1 h.

#### Procedure and stimuli

Participants were seated 20 inches from an 18 inch monitor and provided Sennheiser® headsets. SCR recordings were acquired with BIOPAC System (MP100A-CE) and AcqKnowledge 4.1 recording software. A pair of Ag-AgCl electrodes was attached to the palmar surface of the middle and ring fingers of the participant's dominant hand. Prior to attachment, participants' hands were cleansed with an alcohol wipe and a skin conductance gel was applied to each electrode. SCR was recorded in micro Siemens at a rate of 30 samples/s. Participants were instructed to relax with their dominant hand placed palm up on their thigh and to minimize movement throughout the duration of the experiment. SCR was examined in subjects prior to experimental testing for typicality; absence of a normal response precluded a subjects' participation in the rest of the study.

Stimuli included 31 video clips either acquired from YouTube or recorded in the lab. Video content varied in order to cover a range of sounds and predicted emotional responses in misophonic subjects, selected based on interview data from Experiment 1. Example stimuli included birds singing, children laughing, whale song, nails on a chalkboard, lips smacking, gum chewing, etc. Each clip lasted for 15 s. Auditory and visual components of these videos were separated to generate auditory alone, visual alone, and auditory-visual conditions. Each auditory, visual, and auditory-visual stimulus was presented once for a total of 93 trials. Trial order was randomized into two orders and order was counterbalanced across participants. Critically, as each specific video was presented a total of three times (once in each auditory, visual, and auditory-visual condition), a consistent ordering of the presentation of each stimulus was maintained for each type: auditory alone, visual alone, followed by auditory-visual. Stimuli were presented with E-Prime® version 2.0.

On each trial, participants viewed a centrally presented fixation cross for a 5-s period, followed by either an auditory clip (A), visual movie (V), or auditory-visual movie (AV) for 15 s, concluded with an inter-trial interval of 10 s; during this 10-s interval subjects provided a verbal aversiveness rating on a scale of 0–4 based on how much discomfort they experienced in response to the preceding trial. Participants were informed that a rating of 0 would signify no discomfort at all and a rating of 4 would signify an extreme amount of discomfort, anxiety, or an urge to leave the room. Each aversiveness rating was recorded by the experimenter.

#### Data preprocessing

As our stimuli were presented in quick succession, a linear downward trend was observed throughout the recording session. To account for this artifact, separate linear regressions were fitted to the 5-s fixation period at the start of each trial through a line of best fit. Each observed value during the stimulus epoch was re-plotted as the residual of this line of best fit, normalizing for the pre-stimulus baseline period and removing artifact trends present throughout the epoch. A consistent pattern of results was additionally observed on non-detrended data.

#### Data analysis

SCR onset was time-locked to pre-stimulus fixation cross. Mean SCR was calculated from the 15-s stimulus epoch for each trial, following the fixation cross. Mean values exceeding three standard deviations from the mean SCR across all trials for each participant were deemed outliers and consequently removed from the dataset; an average of 1.9% of trials were removed per participant.

#### Statistical analyses

First, we conducted repeated measures ANOVAs across factors of Group (misophonics, controls), Measurement (SCR, aversiveness rating), and Condition (auditory, visual, auditory-visual) to observe overall effects. Follow-up ANOVAs, non-parametric independent samples tests and descriptive analyses were conducted to explore group differences. Follow-up correlations revealed further group differences as well as similarities. Greenhouse-Geisser corrections were used where appropriate, but we report the original degrees of freedom for clarity.

### Results

#### Overall group effects

As an overall examination of the data, we conducted a repeated measures ANOVA with factors Group (misophonics, controls), Measurement (SCR, subjective rating), and Condition (auditory, visual, auditory-visual). Results showed significant main effects of Group [*F*_(1, 9)_ = 17.5, *p* < 0.005], Condition [*F*_(2, 18)_ = 47.3, *p* < 0.001], and Measurement [*F*_(1, 9)_ = 48.5, *p* < 0.001], as well as significant interactions between Group × Condition [*F*_(2, 18)_ = 18.8, *p* < 0.005], Group × Measurement [*F*_(1, 9)_ = 13.7, *p* < 0.01], Measurement × Condition [*F*_(2, 18)_ = 40.5, *p* < 0.001], and Group × Measurement × Condition [*F*_(2, 18)_ = 16.2, *p* < 0.005].

**Figure 1:**
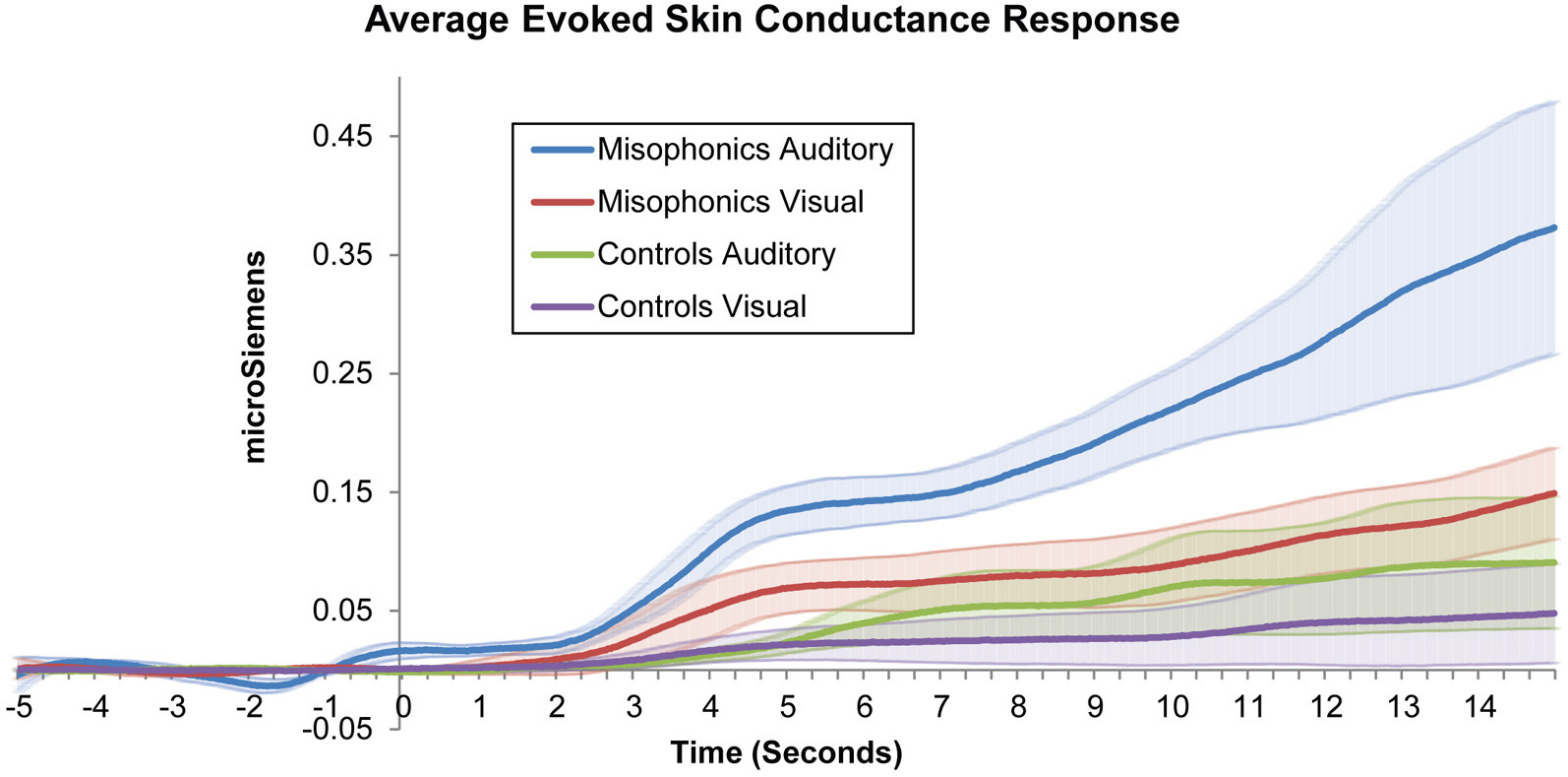
**Average misophonic and control participants' skin conductance response to auditory and visual stimuli as a function of time**.

However, as the primary goal of this study was to examine unisensory responses to stimuli in both groups, subsequent tests for group effects excluded multisensory (auditory-visual) trials and included only auditory and visual conditions. Figure 1 shows misophonic and control subjects' average SCR data in auditory and visual conditions as a function of time. A repeated measures ANOVA with factors of Group (misophonics, controls), Measurement (SCR, subjective rating), and Condition (auditory, visual) similarly identified significant main effects of Group [*F*_(1, 9)_ = 14.3, *p* < 0.005], Condition [*F*_(1, 9)_ = 47.5, *p* < 0.001], and Measurement [*F*_(1, 9)_ = 40.7, *p* < 0.001], as well as significant interactions between Group × Condition [*F*_(1, 9)_ = 17.5, *p* < 0.005], Group × Measurement [*F*_(1, 9)_ = 10.1, *p* < 0.05], Measurement × Condition [*F*_(1, 9)_ = 44.0, *p* < 0.001], and Group × Measurement × Condition [*F*_(1, 9)_ = 16.1, *p* < 0.005]. This overall ANOVA validated the use of follow-up analyses to test specific hypotheses.

#### Group differences

**Figure 2:**
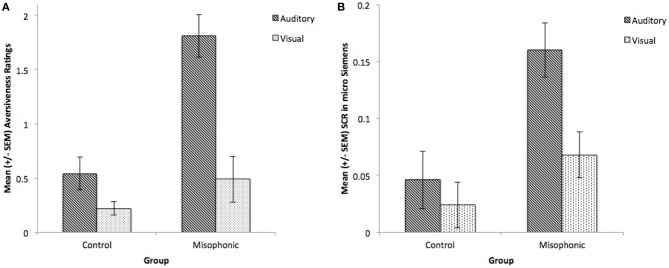
**Group means of controls and misophonics, per presentation condition (auditory and visual) for (A) subjective reports and (B) SCR**.

**Figure 3:**
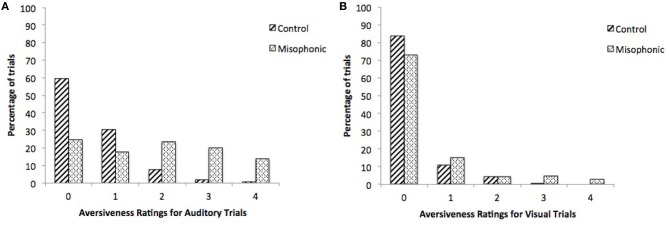
**Percentage of trials per index on the 5-point aversiveness scale, for controls and misophonics, during (A) auditory and (B) visual conditions**.

We conducted additional follow-up repeated measure ANOVAs with factors of Group (misophonics, controls) and Condition (auditory, visual), first for subjective aversiveness ratings alone. Results showed main effects of Group [*F*_(1, 9)_ = 12.4, *p* < 0.01] and Condition [*F*_(1, 9)_ = 46.5, *p* < 0.001], and critically an interaction between the two [*F*_(1, 9)_ = 17.1, *p* < 0.005] supporting the differences between the groups (see Figure 2A). This difference between the groups was largely due to controls rarely rating stimuli as greater than 2 on the aversiveness scale (ranging from 0 to 4; see Figures 3A,B). Examining this model for SCR data yielded a similar pattern of results with main effects of Group [*F*_(1, 9)_ = 6.77, *p* < 0.05] and Condition [*F*_(1, 9)_ = 11.9, *p* < 0.01], and a marginally significant interaction between the two [*F*_(1, 9)_ = 4.53, *p* = 0.06] (see Figure 2B).

Given the small sample size of these groups, follow-up non-parametric independent-samples Mann-Whitney *U*-tests were used to compare groups across these critical conditions. Misophonics reported significantly higher ratings than control subjects in response to auditory stimuli, *U*_(9)_ = 29.0, *p* < 0.01, but not visual stimuli, *U*_(9)_ = 23.5, *p* = 0.13. The median rating of auditory trials was 1.82 (*SD* = 1.38) for misophonics and 0.42 (*SD* = 0.77) for controls while the median rating of visual trials was 0.29 (*SD* = 0.98) for misophonics and 0.19 (*SD* = 0.55) for controls. This pattern of results was consistent with SCR responses, with misophonics producing larger SCR responses than controls to auditory stimuli, *U*_(9)_ = 28.0, *p* < 0.05, but not visual stimuli, *U*_(9)_ = 21.0, *p* = 0.33. The median SCR of auditory trials was 0.15 micro Siemens (*SD* = 0.40) for misophonics and 0.03 micro Siemens (*SD* = 0.11) for controls while the median SCR of visual trials was 0.07 micro Siemens (*SD* = 0.39) for misophonics and 0.00 micro Siemens (*SD* = 0.08) for controls. The same pattern of results for these tests was observed with parametric independent samples *t*-tests.

**Figure 4:**
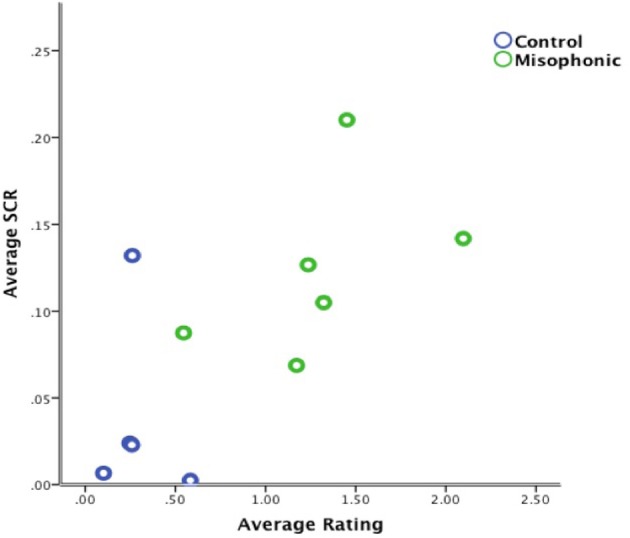
**Correlation of average aversiveness ratings and average SCR (in micro Siemens) for all trials across all subjects**.

In order to determine if higher SCR is directly correlated with higher aversiveness ratings, we examined individual subjects' aversiveness ratings relative to average SCR activity from all auditory, visual, and auditory-visual trials. Results identified a significant positive correlation between average aversiveness ratings and average SCR across all participants (see Figure 4), (*r*_s_ = 0.700, *N* = 11, *Z* = 2.21, *p* < 0.05), indicating that stimuli subjectively thought of as aversive generally evoked a proportional SCR.

#### Group similarities

**Figure 5:**
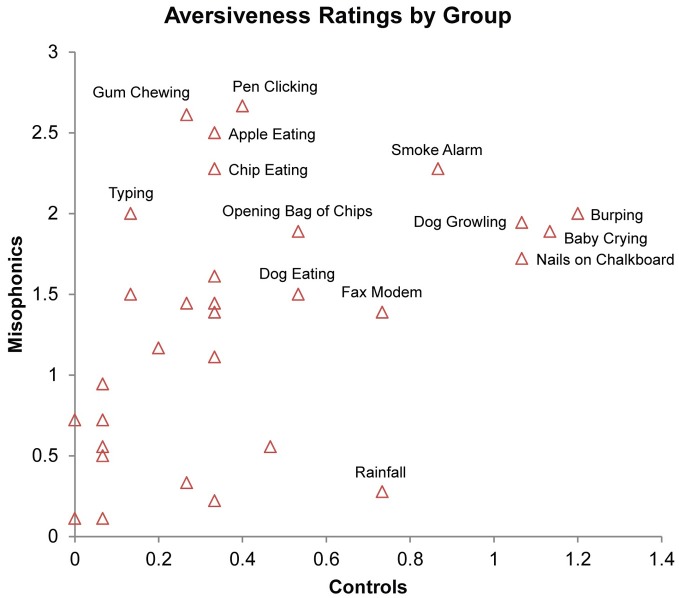
**Correlation of average aversiveness ratings of stimuli (x- and y-axis) across conditions in misophonics and controls.** Select stimuli identified by proximal text.

As an examination of whether the stimuli that trigger aversive experiences in misophonic individuals are idiosyncratic to the condition or consistent to, though more extreme than, preferences present in the general population, we examined the consistency of ratings across the groups. Findings indicated a significant positive correlation between misophonic and control aversiveness ratings across all three types of stimuli, (*r*_s_ = 0.605, *N* = 93, *Z* = 5.80, *p* < 0.001); this correlation is additionally present when examining the correlation between the groups for only auditory trials, (*r*_s_ = 0.413, *N* = 31, *Z* = 2.26, *p* < 0.05; see Figure 5) suggesting that misophonics and controls find similar stimuli to be aversive and non-aversive.

### Discussion

Experiment 2 provides, to the best of our knowledge, the first experimental investigation on misophonia, serving to validate the severity of this chronic condition beyond anecdotal description. Misophonic subjects rated auditory stimuli as more aversive than the same visual stimuli, and this pattern was consistent with SCR measurements. Furthermore, SCR and subjective ratings to auditory stimuli were greater in misophonic individuals than controls, supporting the specificity of aversive reactions in misophonia. Nevertheless, misophonic subjects demonstrated increased ratings and SCR regardless of stimulus type, as revealed by observed main effects of group, possibly denoting generalized anxiety to the stimuli used in the present study.

The significant positive correlation between average aversiveness ratings and mean SCR across all participants importantly confirms the validity of each subject's ratings during the task. Therefore, participant's physiological responses to stimuli were consistent with their subjective ratings. However, as shown in Figure 4, this positive correlation seems most likely driven by group differences between misophonics, (represented in green) and controls (represented in blue).

The significant positive correlation between misophonic aversiveness ratings and control aversiveness ratings reflects a general agreement of the relative valence of the inducing stimuli across the groups. In other words, misophonics and controls find similar stimuli to be aversive and non-aversive on a subjective level, suggesting that misophonics may experience an extreme form of the discomfort most individuals experience to normally aversive or irritating stimuli. This raises the important possibility that there is nothing intrinsically different about misophonic individuals from those in the general population and misophonic individuals are merely at the tail end of the distribution.

## General discussion

In a preliminary examination of individuals with misophonia, we report qualitative and physiological investigations of the condition and its relationship to responses in the typical population. Experiment 1, which is comprised of qualitative assessments on eleven misophonic subjects, examined the qualities associated with misophonia in order to help develop reliable diagnostic criteria and understand the complex social factors involved. Results were consistent with early reports of the phenomenon, such as the critical characteristic of misophonia being a disproportionately aversive reaction is in response to common sounds in everyday life. Additionally, a visceral autonomic response is physically felt in misophonics in response to trigger sounds. In Experiment 2, physiological measurements were acquired on six misophonic individuals using SCR to provide an objective corroboration of misophonics' reports that specific sounds evoke intense emotional reactions. Results showed an increased autonomic response to trigger sounds, but not visual stimuli, in misophonics as compared with non-misophonic controls.

Administering semi-structured interviews proved to be an effective way of determining the most critical symptoms, triggers and experiences associated with misophonia as well as the degree to which these varied across subjects. In addition to reporting psychological symptoms, all of our misophonics reported physical symptoms synonymous with autonomic arousal in response to trigger sounds. Furthermore, our qualitative results are in line with all of the diagnostic criteria proposed by Schröder et al. (2013) which, shortly summarized are: (A) aversive and angry feelings evoked by particular sounds, (B) rare potentially aggressive outbursts, (C) recognition by the misophonic individual that his/her behavior is excessive, (D) avoidance behavior, (E) distress and interference in daily life, and lastly, (F) the lack of another condition to account for all symptoms. Additionally, our principal finding that misophonic individuals experience physical, autonomic arousal that is measurable by SCR, provides empirical validation for some of the aforementioned critical criteria proposed by Schröder et al. (2013), particularly criterion A. Through conducting interviews, we also identified other interesting aspects of misophonia that were not previously apparent. In particular, subjects reported that misophonia can be modulated by social expectations as well as situational context, indicating that the condition may be more complicated than merely an aversive response to the purely physical properties of sounds. Additionally, the finding that misophonic individuals report involuntary, physiological distress in response to a very specific subset of social sounds supplements research on complex mind-body interactions, with high-level knowledge demonstrating prolonged and specific physiological reactions (e.g., as in placebos; Margo, 1999). However, at this time, these speculations remain based on anecdotes and need to be properly tested in the future before firm conclusions can be drawn.

To date, no research has examined the neurological origin of misophonia, and preliminary investigations suggest it is not due to any primary neurological or psychological disorder or trauma (Schröder et al., 2013). Nevertheless, misophonia displays similarities to a genetic condition known as synesthesia. In synesthesia, as in misophonia, particular sensory stimuli evoke particular and consistent, additional sensations and associations. Well-known forms of synesthesia include letters evoking a particular color, or sounds/music evoking colors (Cytowic, 1989; Baron-Cohen et al., 1996; Simner et al., 2006) but there are in fact many different subtypes of synesthesia, with a variety of “inducers” (e.g., music, taste, words, sequences) evoking certain “concurrents” (e.g., color, shapes, taste). While most synesthesia research has examined the perceptual sensations related to synesthesia, the condition seems to have an affective component as well. First, synesthetic congruency (e.g., when a grapheme-color synesthete sees a letter in the “correct” color) is related to positive affect (e.g., Callejas et al., 2007). Furthermore, both inducers (Ward, 2004; Ramachandran et al., 2012) and concurrents (Simner and Holenstein, 2007) can be of emotional rather than perceptual nature. Interestingly, the latter indicates that for certain subtypes of synesthesia, similar to misophonia, inducers evoke a particular feeling or emotion rather than a pure perceptual sensation. This has been studied in tactile-emotion synesthesia (e.g., feeling sandpaper evokes a feeling of jealousy; Ramachandran and Brang, 2008). Synesthetic associations, like misophonic experiences, are automatic (in the sense that they do not take effort or conscious deliberation), are consistent within an individual and persist throughout life, and seem to run in families (Asher et al., 2009; Tomson et al., 2011; for a review see Brang and Ramachandran, 2011). Given these similarities, neuroimaging findings in synesthetes may provide us with hypotheses on the neural basis of misophonia. First, associated sensations in synesthesia are found to be associated with co-activation in relevant (associated) brain areas (Nunn et al., 2002; Hubbard et al., 2005; Rouw et al., 2011). Furthermore, previous studies support a direct linking of relevant sensory regions in synesthesia (Hubbard and Ramachandran, 2001), mediated by an actual increase of anatomical connectivity (Rouw and Scholte, 2007; Zamm et al., 2013). Similarly, altered connections from a lesioned thalamus to the cerebral cortex (Ro et al., 2007; Beauchamp and Ro, 2008) led to a type of acquired synesthesia in which auditory stimuli produced tactile percepts. Differing in the level of specificity and complexity of evoked responses observed in synesthetes, individuals with misophonia display basic and non-elaborated responses to triggering stimuli, varying largely in the intensity of the response. Nevertheless, the underlying neurological cause of this condition may be similar to that of synesthesia in terms of enhanced connectivity between relevant brain regions. In short, a pathological distortion of connections between the auditory cortex and limbic structures could cause a form of sound-emotion synesthesia.

This study also provides the critical finding of a relationship between aversive stimuli in misophonia and mildly aversive stimuli in the general population. That is, in Experiment 2 we observed a significant correlation between aversive ratings across the groups, suggesting that misophonia may be based on mechanisms fundamentally present in the general population, but simply exaggerated in misophonia. Critically, as observed in the interviews in Experiment 1, many of the common aversive stimuli in misophonia are also deemed as socially inappropriate in western society (e.g., lip smacking, repetitive tapping, etc.). While speculative at present, this consistent pattern raises the possibility that the aversive nature of these stimuli to all individuals may be based on the same driving factors (though notably more mild) as in misophonia, leading to the development of these cultural norms.

The present paradigm was designed to include a range of aversive stimuli for misophonic individuals based on our preliminary interviews in Experiment 1. Accordingly, misophonic individuals reported a large number of the stimuli as aversive: mean 24.2% and median 24.7% stimuli with a rating of 3 or 4. In contrast, control participants reported very few stimuli as very aversive: mean 2.4% and median 0.0% stimuli with a rating of 3 or 4 (Figures 3A,B). Potential future studies are suggested to examine if this same pattern of group differences is consistent with stimuli that evoke a broader range of aversive responses in typically developed individuals.

As the current study is exploratory in nature and included a small sample of participants, there are several limitations to acknowledge. One limitation is that the presentation of stimuli in a controlled laboratory setting lacked the ecological validity of how these stimuli occur in the real world. As such, several misophonics reported that because they knew each clip would end in a matter of seconds, their physiological reactions were tempered, consistent with self-reports in Experiment 1 showing that contextual information about these cues mediated subjects' responses. We predict naturalistic observational studies of physiological reactions in misophonic individuals will show a similar but more extreme pattern of results to those observed here. A second limitation is that while SCR is a good measure of autonomic arousal in response to emotion-eliciting stimuli, it does not indicate what specific emotion is being experienced at the time. Instead it only indicates a very general, physiological arousal that can be interpreted in many ways. For example, SCR would not be able to differentiate anxiety and aggression. However, information as to what exactly a subject was feeling during each stimulus can potentially be inferred by obtained self-reports after each trial. A third limitation is the fact that no rigorous diagnostic tests or screenings were utilized during interviews to completely exclude the possibility that subjects' symptoms were being driven by another underlying condition. Also, interviews were conducted by members of our research group and not by psychiatrists. Potentially comorbid conditions were therefore determined from the self-reports of subjects (some of whom had previous, official diagnoses), and the discretion of the researchers. However, because these interviews were not conducted with the intent of being clinical or diagnostic in nature, but rather to gain more insight into the phenomenological experiences of individuals who identify with having misophonia, we believe these findings are still of considerable value to the research community and misophonic individuals alike. A fourth limitation of the study is the small sample size. As research on misophonia is limited to the last few years and little remains known about the condition, obtaining a large sample size for this study was not feasible. Nevertheless, while these results should be validated on a larger group of subjects, we believe they reflect properties of the condition generalizable to the misophonia community in general.

While these data serve to support the veracity of the subjective reports in misophonia as an intrusive and labile condition, numerous additional avenues remain for future research. Critically, as this condition appears to be chronic, the nature of how subjects' triggers evolve over time should be investigated. How does context contribute to and modulate misophonia and can contextual information or expectation effects bias subjects' responses to aversive stimuli? Critically, what are the mechanisms (genetic, neurological, and/or psychological) that underlie the condition? While speculative at present, one potential neural mechanism for misophonia may lie in aberrant anatomical or functional connections between auditory and limbic regions, akin to the finding of increased structural connectivity in synesthesia. Regardless of the mechanisms that underlie misophonia, the present research supports its validity as an intrusive condition and highlights the need for additional research into contributing factors and potential treatments.

### Conflict of interest statement

The authors declare that the research was conducted in the absence of any commercial or financial relationships that could be construed as a potential conflict of interest.

## References

[B1] Asher J. E. Lamb J. A. Brocklebank D. Cazier J. B. Maestrini E. Addis L. (2009). A whole-genome scan and fine-mapping linkage study of auditory-visual synesthesia reveals evidence of linkage to chromosomes 2q24, 5q33, 6p12, and 12p12. Am. J. Hum. Genet. 84, 279–285 10.1016/j.ajhg.2009.01.012 19200526 PMC2668015

[B2] Baron-Cohen S. Burt L. Smith-Laittan F. Harrison J. Bolton P. (1996). Synaesthesia: prevalence and familiarity. Perception 25, 1073–1079 10.1068/p251073 8983047

[B3] Beauchamp M. S. Ro T. (2008). Neural substrates of sound-touch synesthesia after a thalamic lesion. J. Neurosci. 28, 13696–13702 10.1523/JNEUROSCI.3872-08.2008 19074042 PMC6671766

[B4] Brang D. Ramachandran V. S. (2011). Survival of the synesthesia gene: why do people hear colors and taste words. PLoS Biol. 9:e1001205 10.1371/journal.pbio.1001205 22131906 PMC3222625

[B5] Callejas A. Acosta A. Lupiáñez J. (2007). Green love is ugly: emotions elicited by synestheticgrapheme-color perceptions. Brain Res. 1127, 99–107 10.1016/j.brainres.2006.10.013 17112482

[B6] Critcheley H. D. (2002). Electrodermal responses: what happens in the brain. Neuroscientist 8, 132–142 10.1177/107385840200800209 11954558

[B7] Cytowic R. E. (1989). Synaesthesia: A Union of the Senses. New York, NY: Springer-Verlag

[B8] Hadjipavlou G. Baer S. Lau A. Howard A. (2008). Selective sound intolerance and emotional distress: what every clinician should hear. Psychosom. Med. 70, 739–740 10.1097/PSY.0b013e318180edc2 18596245

[B9] Halpern L. Blake R. Hillenbrand J. (1986). Psychoacoustics of a chilling sound. Percept. Psychophys. 39, 77–80 10.3758/BF03211488 3725541

[B10] Hubbard E. M. Arman A. C. Ramachandran V. S. Boynton G. M. (2005). Individual differences among grapheme-color synesthetes: brain-behavior correlations. Neuron 45, 975–985 10.1016/j.neuron.2005.02.008 15797557

[B11] Hubbard E. M. Ramachandran V. S. (2001). Cross wiring and the neural basis of synaesthesia. Invest. Ophthalmol. Vis. Sci. 42, S712 10.1098/rspb.2001.1576 PMC1088697 11370973

[B12] Jastreboff M. M. Jastreboff P. J. (2001a). Components of decreased sound tolerance: hyperacusis, misophonia, phonophobia. ITHS News Lett. 2, 5–7

[B13] Jastreboff M. M. Jastreboff P. J. (2001b). Hyperacusis. Audiology Online. June 2001. Available online at: http://www.audiologyonline.com

[B14] Jastreboff P. J. (2000). Tinnitus habituation therapy (THT) and tinnitus retraining therapy (TRT), in Tinnitus Handbook, ed Tyler R. S. (San Diego, CA: Singular, Thomson Learning), 357–376

[B15] Jastreboff P. J. Hazell J. (2004). Tinnitus Retraining Therapy: Implementing the Neurophysiological Model. New York, NY: Cambridge University Press 10.1017/CBO9780511544989

[B16] Jastreboff P. J. Jastreboff M. M. (2003). Tinnitus retraining therapy for patients with tinnitus and decreased sound tolerance. Otolaryngol. Clin. North Am. 26, 321–336 10.1016/S0030-6665(02)00172-X 12856300

[B17] Kumar S. von Kriegstein K. Friston K. Griffiths T. D. (2012). Features versus feelings: dissociable representations of the acoustic features and valence of aversive sounds. J. Neurosci. 32, 14184–14192 10.1523/JNEUROSCI.1759-12.2012 23055488 PMC3505833

[B18] Margo C. E. (1999). The placebo effect. Surv. Ophthalmol. 44, 33–44 10.1016/S0039-6257(99)00060-0 10466586

[B19] Nunn J. A. Gregory L. J. Brammer M. Williams S. C. R. Parslow D. M. Morgan M. J. (2002). Functional magnetic resonance imaging of synesthesia: activation of V4/V8 by spoken words. Nat. Neurosci. 5, 371–375 10.1038/nn818 11914723

[B20] Ramachandran V. S. Brang D. (2008). Tactile-emotion synesthesia. Neurocase 14, 390–399 10.1080/13554790802363746 18821168

[B21] Ramachandran V. S. Miller L. E. Livingstone M. S. Brang D. (2012). Colored halos around faces and emotion-evoked colors: a new form of synesthesia. Neurocase 18, 352–358 10.1080/13554794.2011.608366 22115465 PMC3630799

[B22] Ro T. Farne A. Johnson R. M. Wedeen V. Chu Z. Wang Z. J. (2007). Feeling sounds after a thalamic lesion. Ann. Neurol. 62, 433–441 10.1002/ana.21219 17893864

[B23] Rouw R. Scholte H. S. (2007). Increased structural connectivity in grapheme-color synesthesia. Nat. Neurosci. 10, 792–797 10.1038/nn1906 17515901

[B24] Rouw R. Scholte H. S. Colizoli O. (2011). Brain areas involved in synaesthesia: a review. J. Neuropsychol. 5, 214–242 10.1111/j.1748-6653.2011.02006.x 21923787

[B25] Schröder A. Vulink N. Denys D. (2013). Misophonia: diagnostic criteria for a new psychiatric disorder. PloS ONE 8:e54706 10.1371/journal.pone.0054706 23372758 PMC3553052

[B26] Schwartz P. Leyendecker J. Conlon M. (2011). Hyperacusis and misophonia: the lesser-known siblings of tinnitus. Minn. Med. 94, 42–43 22413649

[B27] Simner J. Holenstein E. (2007), Ordinal linguistic personification as a variant of synesthesia. J. Cogn. Neurosci. 19, 694–703 10.1162/jocn.2007.19.4.694 17381259

[B28] Simner J. Mulvenna C. Sagiv N. Tsakanikos E. Witherby S. A. Fraser C. (2006). Synaesthesia: the prevalence of atypical cross-modal experiences. Perception 35, 1024–1033 10.1068/p5469 17076063

[B29] Tomson S. Avidan N. Lee K. Sarma A. K. Tushe R. Milewicz D. M. (2011). The genetics of colored sequence synesthesia: suggestive evidence of linkage to 16q and genetic heterogeneity for the condition. Behav. Brain Res. 223, 48–52 10.1016/j.bbr.2011.03.071 21504763 PMC4075137

[B30] Ward J. (2004). Emotionally mediated synaesthesia. Cogn. Neuropsychol. 21, 761–772 10.1080/02643290342000393 21038232

[B31] Zald D. H. Pardo J. V. (2002). The neural correlates of aversive auditory stimulation. Neuroimage 16, 746–753 10.1006/nimg.2002.1115 12169258

[B32] Zamm A. Schlaug G. Eagleman D. Loui P. (2013). Pathways to seeing music: enhancedstructural connectivity in colored-music synesthesia. Neuroimage 74, 359–366 10.1016/j.neuroimage.2013.02.024 23454047 PMC3643691

